# Supplementary material for: Long-Term Iron and Vitamin B12 Deficiency Are Present after Bariatric Surgery, despite the Widespread Use of Supplements
Source: Int J Environ Res Public Health. 2021 Apr 25;18(9):4541. doi: 10.3390/ijerph18094541 (PMC8123142; doi:10.3390/ijerph18094541)
Supplement: Supplementary file 1 [file ijerph-18-04541-s001.zip › ijerph-1133647-supplementary.pdf]

Table S1. Nutritional characteristics of the supplements used.

	Generic Bariatric supplement	RYGB specific supplement	SG specific supplement	Multivitamin supplement	Vitamin D 3 (Cholecalciferol)	Calcium carbonate	Generic vitamin B supplement	Iron (ferrous sulphate)
	Bariatric ©	WLS "Strong" ©	WLS "Optimum" ©	Multicentrum "Adults" ©	DIBASE ©	Natecal ©	Be-Total Plus ©	Ferrograd ©
Iron	30 mg<br>VNR% 214	70 mg<br>VNR% 500	28 mg<br>VNR% 200	5 mg<br>VNR% 36				105 mg<br>VNR% 750
Vitamin D3	25 mcg<br>1.000 IU<br>VNR% 250	75 mcg<br>3.000 IU<br>VNR% 750	75 mcg<br>3.000 IU<br>VNR% 750	10 mcg<br>400 IU<br>VNR% 100	625 mcg<br>25.000 IU<br>VNR% 6250			
Vitamin B12	33 mcg<br>VNR% 1320	350 mcg<br>VNR% 14000	100 mcg<br>VNR% 4000	2.5 mcg<br>VNR% 100			1.5 mcg<br>VNR% 60	
Calcium				162 mg<br>VNR% 20		600 mg<br>VNR% 75		
Amount taken	one tablet per day	one tablet per day	one tablet per day	one tablet per day	*	one tablet per day	one tablet per day	one tablet per day

NRV: 'Nutrient Reference Value' - EU Food Information for Consumers Regulation 1169/2011. * Vitamin D deficient was treated with at least 25,000 IU of Cholecalciferol twice a week to achieve a blood level of 25(OH)D above 30 ng/mL followed by maintenance therapy of 25,000 IU of vitamin D3 once a week.

Table S2. Percent weight loss (%WL) in patients undergoing AGB, SG and RYGB procedures.

				95% Confidence Interval of the Mean		AGB vs SG vs RYGB	SG vs RYGB
Time after surgery (months)	Surgery (n)	Mean (%WL)	Std. Deviation	Lower Bound	Upper Bound	*p* *	*p* #
T3	AGB (11)	15.080	13.3662	5.518	24.642	0.904	
	SG (25)	14.404	7.0153	11.442	17.366		0.323
	RYGB (16)	15.967	11.5310	8.640	23.293		
	Total	14.959	9.6864	12.082	17.835		
T6	AGB (11)	20.775	13.9859	9.083	32.467	0.850	
	SG (24)	22.989	7.1876	19.525	26.454		0.123
	RYGB (15)	23.583	14.0307	14.669	32.498		
	Total	22.718	10.8885	19.188	26.248		
T12	AGB (10)	29.529	21.6162	9.537	49.520	0.017	
	SG (22)	45.652	18.9848	37.011	54.294		0.211
	RYGB (16)	54.208	30.2528	34.987	73.430		
	Total	45.398	24.1657	37.669	53.126		
T24	AGB (11)	20.817	17.0762	2.896	38.737	0.048	
	SG (23)	33.520	10.0871	28.799	38.241		0.166
	RYGB (16)	37.518	15.1987	27.308	47.729		
	Total	32.649	13.7611	28.060	37.237		
T36	AGB (10)	17.600	14.0489	7.550	27.650	0.001	
	SG (24)	32.548	10.0986	28.380	36.716		0.293
	RYGB (16)	36.560	13.7326	28.955	44.165		
	Total	30.762	13.6878	26.872	34.652		
T48	AGB (10)	18.486	14.2470	5.309	31.662	0.011	
	SG (23)	34.513	10.3611	28.776	40.251		0.955
	RYGB (15)	34.325	11.5746	26.971	41.679		
	Total	31.147	13.0359	26.599	35.695		
T60	AGB (11)	20.030	15.1703	9.178	30.882	0.011	
	SG (24)	32.340	11.3465	27.656	37.024		0.891
	RYGB (14)	35.129	11.4821	28.499	41.758		
	Total	30.624	13.1933	26.835	34.414		

T72	AGB (11)	18.770	15.8698	7.417	30.123	0.013	
	SG (24)	32.283	11.6406	27.249	37.316		0.914
	RYGB (15)	33.664	11.9931	26.740	40.589		
	Total	29.819	13.7406	25.785	33.854		
T84	AGB (10)	15.356	14.5924	4.139	26.572	0.005	
	SG (25)	32.068	11.9913	26.752	37.385		0.553
	RYGB (15)	33.092	13.5547	24.479	41.704		
	Total	28.856	14.4970	24.394	33.317		
T96	AGB (11)	11.845	14.3637	2.196	21.495	0.000006	
	SG (25)	31.496	11.3162	26.825	36.167		0.407
	RYGB (16)	34.994	14.2068	27.423	42.564		
	Total	28.415	15.4119	24.125	32.706		

AGB: Laparoscopic Adjustable Gastric Banding SG: Sleeve Gastrectomy. RYGB: Roux-en-y Gastric Bypass. The ANOVA test was performed to compare the % weight loss (%WL) of the three different surgeries over the 8 years (*), with Bonferroni procedure as post-hoc analysis to compare SG and RYGB (#)

Table S3. Number of supplements taken by patients according to gender and surgery.

				**Number of Supplements Used**					**Total**
				**0**	**1**	**2**	**3**	**4**	
F	Surgery	AGB	Count	2	3	2	2	0	9
			% within Surgery	22.2%	33.3%	22.2%	22.2%	0.0%	100.0%
			% within Supplements	25.0%	21.4%	18.2%	25.0%	0.0%	21.4%
			% of Total	4.8%	7.1%	4.8%	4.8%	0.0%	21.4%
		SG	Count	5	3	5	4	0	17
			% within Surgery	29.4%	17.6%	29.4%	23.5%	0.0%	100.0%
			% within Supplements	62.5%	21.4%	45.5%	50.0%	0.0%	40.5%
			% of Total	11.9%	7.1%	11.9%	9.5%	0.0%	40.5%
		RYGB	Count	1	8	4	2	1	16
			% within Surgery	6.3%	50.0%	25.0%	12.5%	6.3%	100.0%
			% within Supplements	12.5%	57.1%	36.4%	25.0%	100.0%	38.1%
			% of Total	2.4%	19.0%	9.5%	4.8%	2.4%	38.1%
	Total		Count	8	14	11	8	1	42
			% within Surgery	19.0%	33.3%	26.2%	19.0%	2.4%	100.0%
			% within Supplements	100.0%	100.0%	100.0%	100.0%	100.0%	100.0%
			% of Total	19.0%	33.3%	26.2%	19.0%	2.4%	100.0%
M	Surgery	AGB	Count	1	1	0	0	0	2

			% within Surgery	50.0%	50.0%	0.0%	0.0%	0.0%	100.0%
			% within Supplements	25.0%	50.0%	0.0%	0.0%	0.0%	20.0%
			% of Total	10.0%	10.0%	0.0%	0.0%	0.0%	20.0%
		SG	Count	3	1	2	1	1	8
			% within Surgery	37.5%	12.5%	25.0%	12.5%	12.5%	100.0%
			% within Supplements	75.0%	50.0%	100.0%	100.0%	100.0%	80.0%
			% of Total	30.0%	10.0%	20.0%	10.0%	10.0%	80.0%
	Total		Count	4	2	2	1	1	10
			% within Surgery	40.0%	20.0%	20.0%	10.0%	10.0%	100.0%
			% within Supplements	100.0%	100.0%	100.0%	100.0%	100.0%	100.0%
			% of Total	40.0%	20.0%	20.0%	10.0%	10.0%	100.0%
Total	Surgery	AGB	Count	3	4	2	2	0	11
			% within Surgery	27.3%	36.4%	18.2%	18.2%	0.0%	100.0%
			% within Supplements	25.0%	25.0%	15.4%	22.2%	0.0%	21.2%
			% of Total	5.8%	7.7%	3.8%	3.8%	0.0%	21.2%
		SG	Count	8	4	7	5	1	25
			% within Surgery	32.0%	16.0%	28.0%	20.0%	4.0%	100.0%
			% within Supplements	66.7%	25.0%	53.8%	55.6%	50.0%	48.1%
			% of Total	15.4%	7.7%	13.5%	9.6%	1.9%	48.1%
		RYGB	Count	1	8	4	2	1	16
			% within Surgery	6.3%	50.0%	25.0%	12.5%	6.3%	100.0%
			% within Supplements	8.3%	50.0%	30.8%	22.2%	50.0%	30.8%
			% of Total	1.9%	15.4%	7.7%	3.8%	1.9%	30.8%
	Total		Count	12	16	13	9	2	52
			% within Surgery	23.1%	30.8%	25.0%	17.3%	3.8%	100.0%
			% within Supplements	100.0%	100.0%	100.0%	100.0%	100.0%	100.0%
			% of Total	23.1%	30.8%	25.0%	17.3%	3.8%	100.0%

AGB: Laparoscopic Adjustable Gastric Banding. SG: Sleeve Gastrectomy. RYGB: Roux-en-y Gastric Bypass. Patients were considered to take the specified supplement if they reported taking that supplement at least 5 days per week. Pearson Chi-Square Test *p* – F= 0.503; M=0.701; Total= 0.434
